# The major depressive disorder GWAS-supported variant rs10514299 in *TMEM161B-MEF2C* predicts putamen activation during reward processing in alcohol dependence

**DOI:** 10.1038/s41398-018-0184-9

**Published:** 2018-07-13

**Authors:** Christine Muench, Melanie Schwandt, Jeesun Jung, Carlos R. Cortes, Reza Momenan, Falk W. Lohoff

**Affiliations:** 1 Section on Clinical Genomics and Experimental Therapeutics, National Institute on Alcohol Abuse and Alcoholism, National Institutes of Health, Bethesda, MD USA; 2 Office of the Clinical Director, National Institute on Alcohol Abuse and Alcoholism, National Institutes of Health, Bethesda, MD USA; 3 Division of Intramural Clinical and Biological Research, National Institute on Alcohol Abuse and Alcoholism, National Institutes of Health, Bethesda, MD USA; 4 Clinical NeuroImaging Research Core, National Institute on Alcohol Abuse and Alcoholism, National Institutes of Health, Bethesda, MD USA

## Abstract

Alcohol dependence (AD) frequently co-occurs with major depressive disorder (MDD). While this comorbidity is associated with an increase in disease burden, worse treatment outcomes, and greater economic costs, the underlying neurobiology remains poorly understood. A recent large-scale GWAS of MDD has identified a locus in the *TMEM161B*-*MEF2C* region (rs10514299) as a novel risk variant; however, the biological relevance of this variant has not yet been studied. Given previous reports of disrupted reward processing in both AD and MDD, we hypothesized that rs10514299 would be associated with differences in striatal BOLD responses during reward/loss anticipation in AD. DNA samples from 45 recently detoxified patients with AD and 45 healthy controls (HC) were genotyped for rs10514299. Participants performed the Monetary Incentive Delay task in a 3-Tesla MRI scanner. Effects of rs10514299 on striatal activation during anticipation of high/low reward/loss were investigated. Furthermore, we examined associations between rs10514299 and lifetime AD diagnosis in two independent clinical samples [NIAAA: *n* = 1858 (1123 cases, 735 controls); SAGE: *n* = 3838 (1848 cases, 1990 controls)], as well as its association with depression severity in a subsample of individuals with a lifetime AD diagnosis *(n* = 953). Patients carrying the T allele showed significantly greater putamen activation during anticipation of high reward (*p* = 0.014), low reward (at trend-level; *p* *=* 0.081), high loss (*p* = 0.024), and low loss (*p* = 0.046) compared to HCs. Association analyses in the NIAAA sample showed a trend-level relationship between rs10514299 and a lifetime AD diagnosis in the European American subgroup (odds ratio = 0.82, *p* = 0.09). This finding was not replicated in the SAGE sample. In the NIAAA sample, the T allele was significantly associated with greater depression symptom severity in individuals with a lifetime AD diagnosis (*β* = 1.25, *p* = 0.02); this association was driven by the African American ancestry subgroup (*β* = 2.11, *p* = 0.008). We show for the first time that the previously identified MDD risk variant rs10514299 in *TMEM161B*-*MEF2C* predicts neuronal correlates of reward processing in an AD phenotype, possibly explaining part of the shared pathophysiology and comorbidity between the disorders.

## Introduction

Alcohol dependence (AD) is a chronically relapsing disorder, characterized by a pattern of compulsive drinking that is maintained despite negative health, social, or occupational consequences. Prevalence rates in the United States of America range from 4.1% in women to 7.8% in men^1^. The etiology of AD is intricate with biological, environmental, and cognitive contributing factors. AD frequently co-occurs with major depressive disorder (MDD). In one study, 20.48% of individuals who met DSM-IV criteria for AD also met criteria for MDD in the previous 12 months, indicating that the odds of MDD are 3.7 times greater for individuals with relative to those without a diagnosis of AD^2^. Subclinical levels of depressive symptoms are experienced by an even larger percentage of patients with AD (80%)^3^. This comorbidity significantly increases the disease burden compared to each of the disorders alone^4^. Moreover, it negatively affects treatment outcomes, which further increases economic losses; therefore, improving our understanding of the genetic underpinnings and neurobiological networks involved is important and may be a crucial step towards the development of novel prevention and treatment strategies^5^.

Both AD and MDD have substantial genetic contributions with heritability estimates of 50–60% for AD and 30–40% for MDD^6–8^. A recent genome-wide association study (GWAS) found a genetic risk variant at the semaphorin 3A locus (*SEMA3A*) to be significantly associated with comorbid AD and MDD in African American but not European American individuals and concluded that further studies are needed to improve our understanding of the genetic underpinnings of this comorbidity^9^. While genetic studies of AD have not been able to identify universal risk variants so far, a recent MDD GWAS using more than 300,000 individuals identified 15 genetic risk loci. The top locus in the joint meta-analysis and the independent replication sample was the single nucleotide polymorphism (SNP) rs10514299 within the *TMEM161B*-*MEF2C* gene cluster. *TMEM161B* encodes the brain-expressed transmembrane protein 161B, and *MEF2C* encodes myocyte enhancer factor 2C, a transcription factor involved in brain development that has been associated with epilepsy, intellectual disability, and hyperkinesis^10–12^; however, it remains unclear what the biological relevance of the rs10514299 variant is.

One prominent clinical feature shared between both AD and MDD is anhedonia, a disruption in the ability to experience and regulate pleasure, which might be modulated by genetic variants^13,14^. Clinical and preclinical research indicates that both disorders are associated with disruptions in the mesolimbic reward system, suggesting that its dysregulation might contribute to the comorbidity of AD and MDD^15,16^. Reward processes are an essential aspect of reward-based learning, specifically, the acquisition of stimulus-action-reward associations, which are crucial for advantageous decision-making and the selection of goal-directed behaviors. The striatum is an important component of the underlying neurocircuitry. Previous research demonstrates that the ventral striatum (i.e., nucleus accumbens) mediates the valuation of rewards and codes for reward prediction errors, while the dorsal striatum (caudate and putamen) appears to be crucial for stimulus-action-reward learning^17–19^. The ability to select appropriate behaviors based on available information about rewards and penalties influences an individual’s adaptability to new situations and whether or not desired outcomes can be achieved. Therefore, there has been substantial interest in neural correlates of the anticipation of rewards/losses in substance use populations, including individuals with AD, where impaired decision-making (e.g., continued use despite adverse consequences) and alterations in sensitivity to rewards and punishments have been shown to contribute to alcohol use initiation, maintenance, and relapse. The Monetary Incentive Delay (MID) task is a widely used and well-validated measure of reward processing in both healthy populations and those with substance use disorders, including AD. Functional neuroimaging studies using the MID task have demonstrated alterations in striatal responsiveness during reward/loss anticipation in individuals with AD^20,21^, as well as MDD^22,23^.

The present study aimed to determine whether genetic variation in rs10514299 is associated with differences in blood-oxygen-level-dependent (BOLD) responses in the striatum (caudate, nucleus accumbens, putamen) during reward/loss anticipation, as assessed by the MID task in individuals with AD compared to healthy controls (HCs). Secondary objectives were to test the hypotheses that genetic variation in rs10514299 would be associated (1) with a lifetime diagnosis of AD in a clinical sample (AD cases and controls) at the National Institute on Alcohol Abuse and Alcoholism (NIAAA Sample), as well as in a sample from the Study of Addiction: Genetics and Environment database (SAGE Sample); and (2) with scores on the Montgomery-Asberg Depression Rating (MADRS) scale in a subsample of the NIAAA Sample that reported a lifetime diagnosis of AD (Lifetime Alcohol Dependence Sample).

## Materials and methods

### Participants

The Neuroimaging Sample included 90 participants (45 AD and 45 HC) with a mean age of 39.78 (*SD* = 11.33; see Table 1). Prior to study participation, participants in the Neuroimaging and NIAAA Sample provided informed written consent in accordance with the Declaration of Helsinki and as approved by the National Institute on Alcohol Abuse and Alcoholism Review Board. All participants completed the Structured Clinical Interview for DSM-IV-TR Axis I Disorders (SCID-IV). Given that DSM-5 criteria for alcohol use disorder are less stringent compared to DSM-IV criteria for AD, all individuals likely fall into the moderate/severe alcohol use disorder categories in DSM-5^24^. Exclusion criteria included left-handedness, contraindications for MRI (e.g., claustrophobia or pregnancy), and the presence of any significant medical or neurological diagnoses as assessed by a history and physical exam. Additional exclusion criteria for the HC group included DSM-IV-TR diagnoses of current/past AD, positive urine drug screens, and alcohol breathalyzer readings above zero. FMRI scans were obtained while participants completed a modified version of the MID task^25^ where approximately 70% of trials were successful. Participants were not on any psychotropic medications on the day of the scan and there were no alcohol abstainers among the HC group. DNA samples were obtained from all participants for genotyping.

**Table 1:** Demographics and characteristics of the Neuroimaging Sample

	Alcohol dependence *N* = 45	Healthy controls *N* = 45	*p* value
Gender			**0.004**
Male	35 (77.8)	22 (48.9)	
Female	10 (22.2)	23 (51.1)	
Age, mean years (SD)	43.25 (10.73)	36.30 (10.94)	**0.003**
Ethnicity			0.088
Black/African American	24 (53.3)	18 (40.0)	
European American	17 (37.8)	16 (35.6)	
Asian	0 (0.0)	6 (13.3)	
Multiracial	1 (2.2)	3 (6.7)	
Unknown	3 (6.7)	2 (4.4)	
Smokers	26 (57.8)	0 (0.0)	**<0.0001**
Rs10514299 Genotype			
CC	35	33	0.624
CT/TT	10	12	
Alcohol dependence severity score, mean (SD)	19.68 (7.70) (*N* = 38)	1.13 (2.25) (*N* = 31)	**<0.0001**
Average number of drinking days in past 90 days, mean (SD)	69.60 (25.19)	18.76 (16.08)	**<0.0001**
Number of heavy drinking days in past 90 days, mean (SD)	59.49 (30.65)	2.42 (7.19)	**<0.0001**
Average number of drinks per drinking day, mean (SD)	13.11 (8.84)	1.80 (1.78)	**<0.0001**
MADRS Score, mean (SD)	12.13 (10.21)	0.87 (1.67)	**<0.0001**
Any anxiety disorder—current	8 (9.0)	1 (1.1)	**0.010**
Any anxiety disorder—lifetime	11 (12.4)	1 (1.1)	**0.002**
Generalized anxiety disorder—current	3 (3.4)	1 (1.1)	**0.030**
Generalized anxiety disorder—lifetime	3 (3.4)	1 (1.1)	**0.030**
Posttraumatic stress disorder—current	2 (2.3)	0 (0.0)	0.150
Posttraumatic stress disorder—lifetime	2 (2.3)	0 (0.0)	0.150
Any mood disorder—current	5 (5.6)	0 (0.0)	**0.020**
Any mood disorder—lifetime	10 (11.2)	8 (9.0)	0.560
Major depressive disorder—current	3 (3.4)	0 (0.0)	0.070
Major depressive disorder—lifetime	9 (10.1)	8 (9.0)	0.750

*Note*. Boldface indicates a significant between-group difference. Numbers reported are *N* (%), unless stated otherwise. Ethnicity reported in this table was based on self-report. Diagnoses are based on DSM-IV-TR and were assessed with the SCID-IV

*MADRS* Montgomery-Asberg Depression Rating Scale

The NIAAA Sample consisted of 1123 individuals with a DSM-IV-TR diagnosis of AD and 735 HCs who enrolled in research studies at the National Institute on Alcohol Abuse and Alcoholism at the National Institutes of Health Clinical Center in Bethesda, MD (see Table 2). 531 individuals with AD and 411 controls were of European American (EA) descent. A total of 507 individuals with AD and 232 controls were of African American (AA) descent. All participants provided blood samples for genotyping.

**Table 2:** Demographics and characteristics of the NIAAA Sample and the Lifetime Alcohol Dependence Sample

	NIAAA Sample *N* = 1858		NIAAA subsample with lifetime AD
	Cases *N* = 1123	Controls *N* = 735	*N* = 955
Gender, *N*, (%)			
Male	323 (28.8)	325 (44.2)	669 (70.0)
Female	800 (71.2)	410 (55.8)	286 (30.0)
Age, mean years (SD)	42.6 (10.9)	32.8 (11.6)	42.8 (10.8)
Ethnicity, *N*, (%)			
Black/African American	507 (45.2)	232 (31.6)	439 (46.0)
European American	531 (47.3)	411 (55.9)	441 (46.1)
Asian	16 (1.4)	47 (6.4)	12 (1.3)
Multiracial	21 (1.9)	20 (2.7)	18 (1.9)
Native American or Alaska Native	5 (0.5)	2 (0.3)	5 (0.5)
Native Hawaiian or other Pacific Islander	1 (0.1)	1 (0.1)	1 (0.1)
Unknown	42 (3.7)	22 (3.0)	39 (4.1)
Smokers, *N*, (%)	662 (59.0)	44 (6.1)	595 (62.3)
MADRS score, mean (SD)	13.2 (9.8)	1.3 (3.2)	13.2 (9.8)
Major depressive disorder—current, *N*, (%)	108 (5.8)	8 (0.4)	103 (10.8)
Major depressive disorder—lifetime, *N*, (%)	216 (11.6)	49 (2.6)	196 (20.5)

*Note*. Ethnicity reported in this table was based on self-report

*MADRS* Montgomery-Asberg Depression Rating Scale

The SAGE was funded by the National Human Genome Research Institute as part of the Gene Environment Association Studies (GENEVA) that consisted of three major studies of addictive disorders: The Collaborative Study on the Genetics of Alcoholism (COGA), the Family Study of Cocaine Dependence (FSCD), and the Collaborative Genetic Study of Nicotine Dependence (COGEND). The accession number of dbGaP is phs000092.v1.p1. The EA subgroup of the SAGE Sample consisted of 1162 cases (mean age = 38.2 (9.94); 62% male) and 1346 controls (mean age 38.6 (9.44); 30.3% male). The AA subgroup consisted of 686 cases (mean age = 40.3 (7.7); 61% male) and 644 controls (mean age = 39.6 (7.2); 36% male). All cases met lifetime DSM-IV criteria for AD, and were excluded if they had schizophrenia or any other psychotic illness. Controls were defined as individuals who had been exposed to alcohol, but had not met lifetime criteria for AD or other illicit substances.

### Experimental paradigm

Participants completed a modified version of the MID task^25^, which required a fast button response to a target to win or avoid losing money. A trial started with a crosshair for fixation, then a 2-second presentation of a cue (a shape indicating the type of trial: reward, loss, or neutral). After a delay screen of variable duration (between 0.75 and 1.75 s) the target was presented (a white-filled square to which the participants were trained to respond to). Next, another delay screen of variable duration (between 0.75 and 3 s) was presented before the feedback screen appeared. The feedback screen showed the amount that was won or lost in the previous trial, which represented if the participant had successfully pressed the button in time to win or avoid losing the target amount. The total amount of money accumulated up to that point was also presented on the feedback screen.

After practicing outside of the scanner and being informed that gains/losses during the task would be reflected in their final payment, each subject completed a 12-minute fMRI session of the MID task. Five different cues were presented thirty times, each representing a different trial condition; neutral (no reward/no loss), low loss, high loss, low reward, and high reward.

### MRI data acquisition and preprocessing

A Siemens 3 T Skyra scanner was used to collect whole-brain echo-planar imaging functional (36 axial slices, 4 mm slice thickness, 64 × 64 matrix and repetition time of 2000 ms) and MPRGE structural data. Preprocessing and analysis was performed using AFNI software package^26^. Large transients removal was performed before slice-time correction, then nuisance variables were de-trended, including the six parameter estimates for head motion. No participants had any condition censored more than 20% (motion higher than 0.3 mm was censored out), a criterion to be excluded from the analysis. Images were then coregistered to the individual structural image, resampled (2-mm isotropic), smoothed (4-mm full-width-half-maximum Gaussian kernel), normalized by the mean signal intensity in each voxel to reflect percent signal change, and finally transformed into the standardized^27^.

### BOLD fMRI data and ROI analysis

Single-subject level analysis was performed using AFNI^26^. Then, using ROI masks of the bilateral caudate, putamen, and nucleus accumbens, generated with AFNI *DrawDataset* plugins and following previously published literature and subtracting all nuances effects (such as pre-motor activations), except reward/loss response, we computed average percent signal change (beta weights) of the following comparisons: high reward > neutral, low reward > neutral, high loss > neutral, low loss > neutral^28,29^. For simplification purposes, we refer to these as high reward, low reward, high loss, and low loss, respectively.

### Genotyping

#### NIAAA Sample

Large-scale genotyping was performed at the NIAAA Laboratory of Neurogenetics using the Illumina OmniExpress BeadChip (Illumina, San Diego, CA). Data for the SNP rs10514299 located within the *TMEM161B-AS1* gene cluster was extracted from the larger array. Ancestry informative markers (AIMs; *n* = 2500) were extracted from the Illumina array to calculate ancestral proportions for all study participants. Using methods described previously for an AIM panel including 186 markers^30^, which were not available for the current data set, the ancestry assessment identified 6 ethnic factors (Africa, Europe, Asia, Far East Asia, Oceania, and Americas).

#### SAGE Sample

Whole-genome genotyping was completed using the Illumina Human-1M platform (Illumina Inc., San Diego, CA). The 190 HapMap samples (48 HapMap control samples, 55 CEU, 55 YUR, 13 CHB, and 17 JPT) ^31^ were genotyped in the same platform and population stratification analyses were performed using principal component analysis. After a series of quality control procedures (*p* value of Hardy-Weinberg equilibrium (HWE) < 10e-5, missing rate by SNP > 2%, missing rate by individual > 2%, minor allele frequency < 1%, sex inconsistency), there were 1162 case and 1346 control samples in the EA subgroup and 686 case and 644 control samples in AA subgroup. Two population stratification scores (PSS) calculated based on principal component analysis were used for statistical analyses.

### Statistical analysis

Main analyses of the Neuroimaging Sample were run using SPSS statistical software package (IBM SPSS Statistics ® 20, IBM Corp. ©, Armonk, NY). Baseline differences in demographic variables between cases and controls were determined using t-tests for continuous and chi-square tests for categorical variables. Chi-square tests were performed to test the genotypic distribution of the SNP in both cases and controls. The primary outcome measure was activation in the caudate, putamen, and nucleus accumbens during reward/loss anticipation as measured by BOLD fMRI. Shapiro-Wilk tests confirmed that all four imaging outcomes (high reward, low reward, high loss, low loss) were normally distributed (all *p* ≥ 0.407). For the main analysis of effects of diagnosis, genotype, and their interaction on BOLD responses in the caudate, putamen, and nucleus accumbens, we conducted 2 × 2 analyses of covariance (ANCOVAs) with group (AD vs. HC) T allele carrier status (CC vs. TT/CT–assuming a dominant model) as factors and age, gender, AIM score Europe, and AIM score Africa as covariates. All tests were two-tailed and considered to be statistically significant at *p* *<* 0.05.

For the NIAAA Sample, minor allele frequency, HWE, and association analyses were conducted using PLINK version 1.07. Analyses were conducted first in the entire sample, and then separately for EA and AA subgroups, based on self-report. Case-control association with lifetime AD was determined using logistic regression, while association with the MADRS scale was determined using linear regression. Both analyses used an additive model and adjusted for age, gender, and AIM scores for European and African ancestry. Similarly, for the SAGE Sample, a logistic regression model controlling for age, gender, and 2 PSS for African and European ancestry was performed with the assumption of an additive model. Analyses were performed separately in individuals of European and African ancestry.

## Results

### Neuroimaging Sample

Chi-square goodness-of-fit tests demonstrated that none of the genotype counts deviated significantly from the HWE both in the whole sample (*p* = 0.657), as well as in the subgroups (AD: *p* *=* 0.649; HC: *p* = 0.302).

**Fig. 1:**
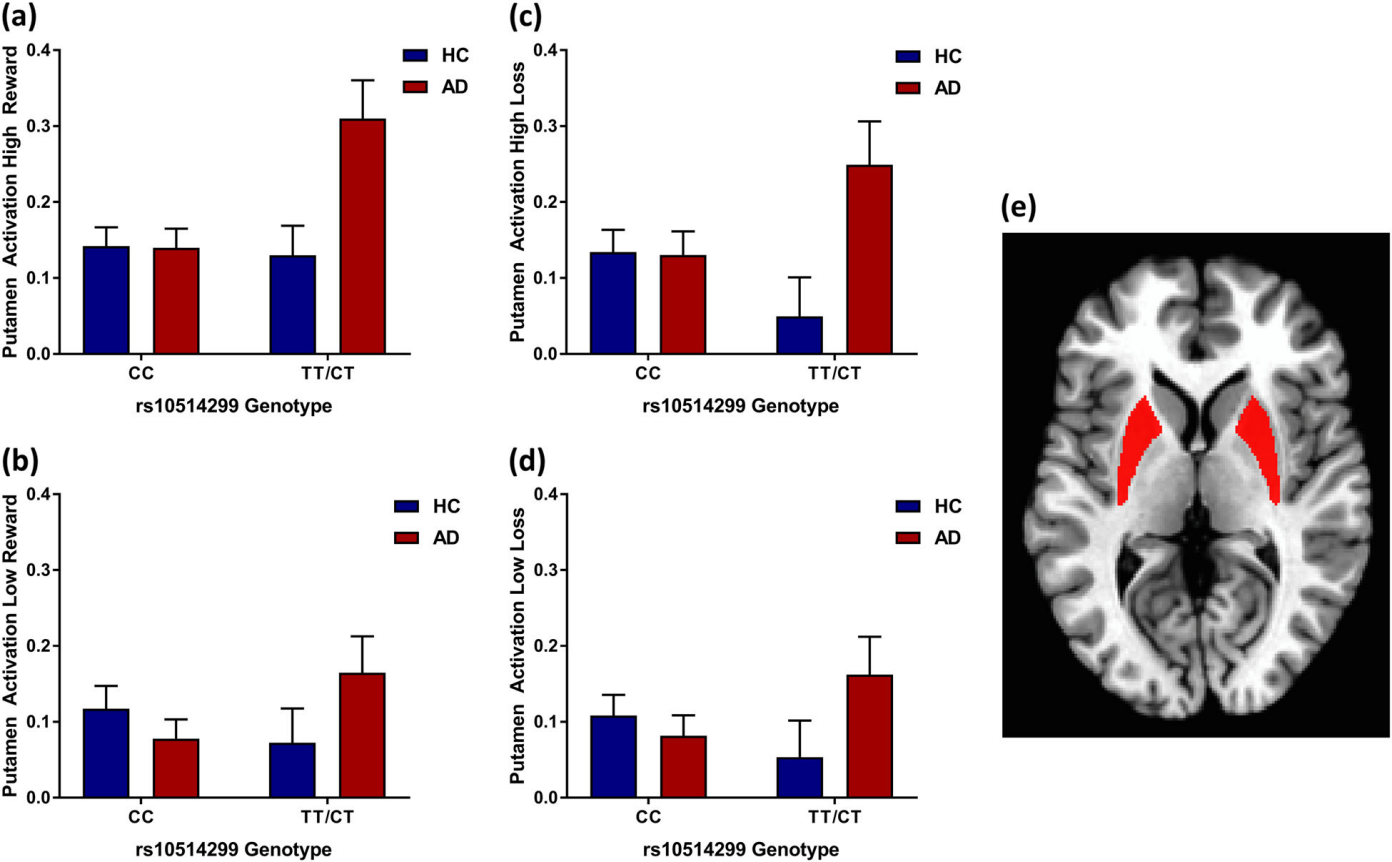
Interaction effects of rs10514299 and alcohol dependence on putamen activation. Bar graphs of the interaction between rs10514299 genotype and alcohol dependence diagnosis on putamen activation during (**a**) high reward anticipation; (**b**) low reward anticipation; (**c**) high loss anticipation; and (**d**) low loss anticipation. Error bars represent standard error of the mean. (**e**) Putamen mask (displayed at *z* = 5) obtained from Talairach Daemon Atlas in AFNI used to extract individual subjects’ beta coefficients for reward/loss anticipation. AD alcohol-dependent group, HC healthy control group

For the main analyses, effects of diagnosis (AD vs. HC), rs10514299 genotype (CC vs. TT/CT), and their interaction on striatal activation were examined using 2 × 2 ANCOVAs controlling for age, gender, AIM Africa, and AIM Europe. Parameter estimates can be found in Table 3. Levene’s test and normality checks were carried out and assumptions were met. There were no significant group-by-genotype findings for the caudate and nucleus accumbens (see Supplementary Table 1). However, results showed a significant diagnosis-genotype interaction effect (*F*(1,82) = 6.37, *p* = 0.014, *η*_*p*_^*2*^ = 0.07) on putamen activation during high reward anticipation. In the control group, both genotype groups showed similar putamen activation during high reward anticipation; however, in the AD group, individuals who carry the minor allele T (TT/CT) showed significantly greater putamen activation during high reward anticipation compared to CC carriers (see Fig. 1a). Furthermore, there was a trend-level diagnosis-genotype interaction effect (*F*(1,82) = 3.12, *p* = 0.081, *η*_*p*_^*2*^ = 0.04) on BOLD activation in the putamen during the anticipation of low reward. Carrying the T allele was associated with decreased putamen activation during low reward anticipation in the HCs, while it was associated with increased putamen activation during low reward anticipation in individuals with AD (see Fig. 1b). Additionally, there was a significant diagnosis-genotype interaction effect (*F*(1,82) = 5.25, *p* = 0.024, *η*_*p*_^*2*^ = 0.06) on BOLD activation in the putamen during the anticipation of high loss. Carrying the T allele was associated with significantly decreased putamen activation during high loss anticipation in the HCs, while it was associated with significantly increased putamen activation in individuals with AD (see Fig. 1c). Similarly, there was a significant diagnosis-genotype interaction effect (*F*(1,82) = 4.10, *p* = 0.046, *η*_*p*_^*2* *=* ^0.05) on putamen activation during low loss anticipation. In the HC group, T allele carriers showed significantly lower putamen activation, whereas individuals with AD showed significantly greater putamen activation during low loss anticipation (see Fig. 1d).

**Table 3:** Parameter estimates of main analysis ANCOVAs examining effects of group, rs10514299 genotype, and their interaction on putamen MID task percent signal change controlling for age, gender, and ancestry informative markers

Putamen activation	B	Standard error	*t*	*p* value	Partial eta squared
High reward					
Group	0.21	0.06	−3.34	**0.001**	0.12
MA (T) carrier	0.15	0.05	−2.92	**0.005**	0.09
Interaction	0.18	0.07	2.52	**0.014**	0.07
Low reward					
Group	0.14	0.07	−1.94	0.055	0.04
MA (T) carrier	0.08	0.06	−1.38	0.173	0.02
Interaction	0.14	0.08	1.77	0.081	0.04
High loss					
Group	0.24	0.08	−3.15	**0.002**	0.11
MA (T) carrier	0.1	0.06	−1.59	0.115	0.03
Interaction	0.2	0.09	2.29	**0.024**	0.06
Low loss					
Group	0.17	0.07	−2.49	**0.015**	0.07
MA (T) carrier	0.08	0.06	−1.46	0.148	0.03
Interaction	0.16	0.08	2.03	**0.046**	0.05

*Note*. *MA (T*) minor allele T

Boldface indicates significance

### NIAAA Sample

The minor allele T frequency of rs10514299 was 0.21 in the full sample (*n* = 1858), 0.25 in subjects of European ancestry (*n* = 942), and 0.18 in subjects of African ancestry (*n* = 739). HWE was not violated in the full sample *(p* = 0.33), nor in the EA (*p* = 0.10) or AA (*p* = 0.53) subgroups.

Case-control analysis revealed a trend association between rs10514299 and lifetime diagnosis of AD but only in the EA subgroup (Table 4). In this sample, the minor allele T was associated with a protective effect against lifetime AD (odds ratio = 0.82, *p* = 0.09).

**Table 4:** Results of association analyses in the NIAAA Clinical Sample

	Lifetime alcohol dependence						MADRS score^a^		
Sample	Cases (*n*)	Controls (*n*)	Cases MAF	Controls MAF	Odds ratio	*p* value	Sample (*n*)	Beta	*p* value
All subjects	1123	735	0.21	0.22	0.95	0.45	955	1.25	**0.02**
European/Caucasian^b^	531	411	0.23	0.28	0.82	0.09	442	0.85	0.27
African American^b^	507	232	0.18	0.17	1.07	0.67	439	2.10	**0.008**

*Note*. *MAF* minor allele T frequency, *MADRS* Montgomery-Asberg Depression Rating scale

^a^Only in subjects with lifetime alcohol dependence. Boldface indicates significance.

^b^Based on self-report

Among subjects reporting a lifetime AD diagnosis, there was a significant association between rs10514299 and the MADRS score in the full sample, as well as in the AA subgroup (Table 4). Specifically, the minor allele T was associated with an increased MADRS score (full sample: *β* = 1.25, *p* = 0.019; AA subgroup: *β* = 2.10, *p* = 0.008).

### SAGE Sample

The minor allele T frequency of rs10514299 was 0.23 in the full sample (*n* = 3838), 0.24 in subjects of European ancestry (*n* = 2508), and 0.18 in subjects of African ancestry (*n* = 1330). Genotypes did not deviate from HWE. There was no significant association of the T allele with AD in either the EA sample (1162 cases, 1346 controls; odds ratio *=* 0.97*, p* = 0.700) or the AA sample (686 cases, 644 controls; odds ratio *=* 1.06*, p* = 0.560).

## Discussion

The present study investigated the influence of a recent GWAS-identified risk variant in the *TMEM161B*-*MEF2C* gene cluster for MDD on striatal activation during the anticipation of high/low rewards/losses in individuals with AD and HCs. Furthermore, we examined associations between genetic variation in rs10514299 and a lifetime AD diagnosis in two clinical samples, and its association with depressive symptom severity as measured by the MADRS in a subsample of individuals with a lifetime AD diagnosis. Our data show for the first time an association of rs10514299 with a neuronal phenotype, namely putamen activation during a reward task. We found that patients with AD who carry the minor allele T showed significantly greater putamen activation during the anticipation of high reward compared to controls (Table 3; Fig. 1a). Furthermore, carrying the T allele was associated with a significant increase in putamen activation during high and low loss anticipation in patients with AD, but with a significant decrease in the controls, indicating that the minor allele T differentially affects this neural phenotype in AD (Table 3; Fig. 1c, d). These data are in line with prior studies that have shown disruptions in the dorsal striatum in individuals with AD during reward anticipation. e.g.,^32^. Neuroimaging studies in humans and neural recording studies in monkeys indicate that the putamen and caudate are involved in different aspects of reward-based learning e.g.,^19,33^. While the caudate is involved in the coding of prediction errors, the putamen has been shown to mediate stimulus-action-reward associations^17^. Our present study suggests that genetic variation in rs10514299 within the *TMEM161B*-*MEF2C* gene cluster may serve as a biomarker for disrupted reward processing in the putamen in alcohol-dependent individuals. Future studies are needed to further investigate the functional relevance of this SNP in individuals with AD and to examine its association with the behavioral impairments in decision-making and reward-based learning that are often observed in AD populations. Furthermore, future studies should include seed-based correlation analyses to investigate the functional connectivity between reward-associated structures of the striatum (caudate, putamen, nucleus accumbens) and associated brain regions.

Our finding is intriguing given the often observed deficit of reward processing in MDD^22,23^ that is described clinically as anhedonia^34^. Similar to other psychiatric disorders, current DSM-5 nosology for mood disorders and substance use disorders is only descriptive and does not capture the underlying neurobiological pathophysiology. The fact that a MDD risk variant was also shown to have an effect in an AD neuroimaging sample supports a potential role of this genetic variant in an endophenotype related to reward processing that might cross DSM-IV/DSM-5 categories. Myocyte enhancer factor 2C is a transcription factor that has been shown to be involved in neurodevelopment, hippocampus-dependent learning, and memory^35^. Prior studies have shown *MEF2C* haploinsufficiency, deletions, and point mutations to be associated with seizures, cortical malformations, intellectual disability, and hyperkinesis^11,12,36–39^. *TMEM161B* is largely uncharacterized and the present study is one of the first to identify its potential functional relevance for a neuronal phenotype in a clinical population. Future clinical and preclinical studies should further investigate the relevance of this gene for anhedonia-related symptomatology in psychiatric disorders, such as substance use disorders, schizophrenia, MDD, and their co-occurrence to dissect potential genetic effects on reward circuitry.

To further investigate the role of this MDD risk variant in AD, we conducted association analyses in two independent clinical samples. In the NIAAA Sample, we found a trend-level association between rs10514299 and a lifetime diagnosis of AD in the EA subgroup only, where the minor allele T appeared to have a protective effect. A replication attempt using the larger SAGE Sample (*n* *=* 3838) failed to replicate this association, suggesting that the effect size of the risk variant in AD is relatively small. This is consistent with the prior GWAS in MDD of 300,000 samples, which reported a rather small effect size (OR = 1.05)^10^. Other potential reasons for the lack of replication are presence of confounding factors, such as clinical heterogeneity as individuals with a lifetime diagnosis of AD are more likely to present with comorbid psychiatric diagnoses and other substance use disorders. Interestingly, our data showed a marginally significant protective effect of rs10514299 for AD, while Hyde et al.^10^ identified the minor allele T as conferring risk for MDD. It is possible that the T allele is a risk allele for MDD and a protective allele for AD, especially given the fact that both disorders present with highly heterogeneous clinical phenotypes. However, it should be noted that a more recent GWAS of 130, 664 MDD cases and 330, 470 HCs did not replicate rs10514299 as a risk variant for MDD^40^, suggesting the possibility that the signal may depend on the presence of other variants or may be driven by other SNPs altogether. Future GWAS with large sample sizes are needed to further elucidate the relevance of rs10514299 and proximal SNPs for an increased or reduced risk for MDD and AD.

To investigate the effect of this SNP on depressive symptoms in AD, we looked at the MADRS in a subsample of the NIAAA Sample. Our data showed that the minor allele T of rs10514299 was significantly associated with greater severity of depressive symptoms in individuals with a lifetime AD diagnosis (Table 4). This association was mainly driven by cases of AA ancestry; however, our sample might have been too small to detect effects across ethnic backgrounds. Furthermore, it should be noted that the medication status for this sample was not available. Given that antidepressant medication may influence the relationship between rs10514299 and depressive symptoms, this finding should be replicated in future studies that assess and control for psychotropic medication status. Importantly, future studies are needed to further investigate this relationship and to examine whether minor allele T carrying individuals with AD might particularly benefit from treatments that focus on negative affect relief, such as mindfulness-based interventions, or antidepressant medications. If future research confirms this variant as a biomarker of an increased risk for depressive symptomatology in individuals with AD, it may be used by clinicians for the selection of more individualized interventions.

When interpreting the findings from the Neuroimaging Sample, the following limitations should be considered. This was a discovery study that aimed to explore genetic variation in rs10514299 as a possible biomarker for interindividual differences in reward-related neuronal activation in individuals with AD. Therefore, we did not correct for multiple comparisons. However, future confirmatory analyses will be done to validate the functional relevance of rs10514299 in neuroimaging phenotypes in alcohol-dependent populations. Additionally, cases and controls were not well matched for age and gender, therefore, these variables were included as covariates in all analyses. Cases and controls differed significantly in smoking status, however, all results remained significant when analyses were repeated while controlling for smoking status.

Limiting factors for the case-control association analyses include small sample sizes and thus a lack of statistical power to detect the likely small effect size of this SNP as discussed above. We only focused on one variant but this variant was initially identified in a cluster of SNPs. Future studies should investigate possible other important functional variants, such as nearby SNPs with strong linkage disequilibrium by sequencing all promising regions. Both AD and MDD are highly heritable and influenced by polygenic effects associated with many genetic variants, and therefore genetic interaction among the many genes related to the single variant on the same pathways should be examined functionally^41^.

In summary, our data suggest an association between rs10514299 and BOLD activation in the putamen during reward and loss anticipation in individuals with AD. Carrying the minor allele T differentially affects putamen activation during reward/loss anticipation in individuals with AD compared to HCs, indicating that genetic variation in rs10514299 may be a clinical biomarker for a neuronal phenotype in AD. Furthermore, the minor allele T was significantly associated with greater levels of depressive symptoms in individuals with a lifetime diagnosis of AD, a finding that was driven by individuals of African American ancestry. Finally, association analyses showed a relationship at trend-level between rs10514299 and a lifetime diagnosis of AD in European Americans in the NIAAA Sample but not in the SAGE Sample. This is the first study to show functional relevance of rs10514299 for an imaging phenotype in individuals with AD, as well as its relevance for depressive symptom severity in individuals with a lifetime diagnosis of AD. Taken together, our findings indicate rs10514299 as a promising target for future study in populations with AD and MDD. Identifying genetic risk variants may serve to guide the selection of treatment options, as well as reveal novel treatment targets, while neuronal phenotypes may serve as markers of treatment response to cognitive, behavioral, or pharmacological interventions. Ultimately, the identification of objective biomarkers of disease-related mechanisms will contribute to the development of individualized treatments.

## Electronic supplementary material

Supplementary Table 1
