# Supplementary material for: The major depressive disorder GWAS-supported variant rs10514299 in TMEM161B-MEF2C predicts putamen activation during reward processing in alcohol dependence
Source: Transl Psychiatry. 2018 Jul 13;8:131. doi: 10.1038/s41398-018-0184-9 (PMC6045574; doi:10.1038/s41398-018-0184-9)
Supplement: Supplementary file 1 — Supplementary Table 1 [file 41398_2018_184_MOESM1_ESM.docx]

**Supplementary Materials Muench et al.**

Supplementary Table 1. Results of ANCOVAs examining effects of group, rs10514299 genotype, and their interaction on caudate and nucleus accumbens MID task percent signal change controlling for age, gender, and ancestry informative markers.

|  | Caudate | | Nucleus Accumbens | |
| --- | --- | --- | --- | --- |
|  | F | P-Value | F | P-Value |
| High Reward  Group  MA (T) Carrier  Interaction  Low Reward  Group  MA (T) Carrier  Interaction  High Loss  Group  MA (T) Carrier  Interaction  Low Loss  Group  MA (T) Carrier  Interaction | 8.02  3.99  2.92  1.95  0.22  .062  7.24  0.01  1.39  2.00  0.01  0.34 | **.006**  **.049**  .091  .166  .641  .434  **.009**  .934  .242  .161  .924  .560 | 3.47  2.32  0.07  6.13  0.02  1.72  5.59  0.06  0.00  3.95  0.82  0.05 | .066  .132  .782  **.015**  .880  .194  **.020**  .802  1.00  .050  .368  .832 |

*Note.* MA (T) = minor T allele. Boldface indicates significance.
